# Transcriptomic Profiling of the Effects of DDR1 in Breast and Ovarian Cancer to Understand the Association Between DDR1 Expression and Patient Survival

**DOI:** 10.3390/genes17070760

**Published:** 2026-06-30

**Authors:** Khalid Alshammari, Suha Deen, Ian O. Ellis, Emad A. Rakha, Andrew R. Green, Stewart G. Martin, Sarah J. Storr

**Affiliations:** Nottingham Breast Cancer Research Centre, School of Medicine, University of Nottingham Biodiscovery Institute, University Park, Nottingham NG7 2RD, UK; alyka16@nottingham.ac.uk (K.A.); sdeen2014@live.co.uk (S.D.); ian.ellis@nottingham.ac.uk (I.O.E.); andrew.green@nottngham.ac.uk (A.R.G.); stewart.martin@nottingham.ac.uk (S.G.M.)

**Keywords:** DDR1, breast cancer, ovarian cancer

## Abstract

**Background:** Discoidin domain receptor 1 (DDR1) is a collagen-activated receptor tyrosine kinase that plays an important role in epithelial cell regulation; its function in cancer appears to be dependent on tumour type. **Methods:** This study investigated DDR1 expression in large numbers of breast (*n* = 1416) and ovarian (*n* = 450) tumours using immunohistochemistry. In addition, RNA sequencing was conducted on *DDR1* knockdown breast and ovarian cancer cell lines. **Results:** In breast cancer, high DDR1 expression was significantly associated with poor patient survival in ER-positive disease and low expression was associated with poor patient survival in ER-negative disease. In ovarian cancer, high DDR1 expression was associated with improved patient survival. In *DDR1* knockdown IGROV1 ovarian cancer cells, 770 transcripts were differentially expressed, whilst in *DDR1* knockdown T47D breast cancer cells, 3647 transcripts were differentially expressed. Only 149 genes were shared, suggesting that DDR1 drives distinct transcriptional programmes across cancer types. Shared genes between T47D and IGROV1 *DDR1* knockdown cells include key regulators of signalling, metabolism, and cytoskeletal organisation such as *YWHAE*, *NCK2*, *FN1*, and *ITGB4*. Gene Ontology analysis revealed significant enrichment of epithelial cell migration pathways in both cell lines. **Conclusions:** Current protein expression and transcriptomic data highlight the important prognostic role of DDR1 expression in breast and ovarian cancer and provide hypothesis-generating insights into the contextual and transcriptomic differences between the two cancer types.

## 1. Introduction

Discoidin domain receptors (DDRs) are a subfamily of receptor tyrosine kinases (RTKs) that are characterised by a discoidin homology domain in their extracellular regions. These receptors were originally identified in 1993, with molecular characterisation in 1997, including description of their activation by collagen resulting in slow but persistent kinase activity [1,2]. The human DDR1 gene is located on chromosome 6 and comprises 17 exons, with alternative splicing resulting in five distinct protein isoforms. Among these, DDR1a, DDR1b, and DDR1c are full-length, functional receptors, whereas DDR1d and DDR1e lack kinase activity [3]. Structurally, DDR1 is a one-way transmembrane receptor consisting of six structural domains: the N-terminal extracellular discoidin domain (DS); the discoidin-like (DSL) domain, which is responsible for collagen binding; the extracellular juxtamembrane region (EJXM); a single transmembrane segment (TM); an intracellular juxtamembrane region (IJXM); and a C-terminal kinase domain (KD) [4]. Evidence indicates that, once activated, DDR1 initiates signalling cascades (e.g., MAPK, PI3K/AKT, and NF-κB) that regulate multiple cellular processes, including cell adhesion, migration, proliferation, survival, and extracellular matrix remodelling, supporting its role as an important modulator of epithelial cell behaviour [5].

Aberrant expression of DDR1 was reported in oesophageal cancer as early as 1997, and in high-grade brain neoplasms in 2000, with further pan-cancer studies demonstrating that DDR1 expression is dysregulated across multiple cancer types [6,7,8]. In breast cancer, DDR1 was initially shown to have an essential role in mammary gland development, where it mediated stromal–epithelial interactions during ductal morphogenesis [9], and more recently, DDR1 was shown to direct stem cell differentiation into basal cells, which subsequently stimulated luminal progenitor cells to form lobules via Notch signalling [10]. Beyond development, DDR1 is implicated in the regulation of breast cancer cell migration in vitro. Wnt-5A has been shown to enhance collagen-induced DDR1 activation, thereby restricting cell migration, and this effect can be regulated by TGF-β signalling [11,12]. In a separate study, DDR1 was demonstrated to suppress breast cancer cell migration through the formation of a functional complex with DARPP-32, as DARPP-32 impaired migration only in the presence of DDR1, indicating that DARPP-32 acts downstream of DDR1 signalling [13]. Further work demonstrated that Wnt-5A can also induce a DARPP-32-dependent antimigratory response via activation of a Frizzled-3/Gαs/cAMP/PKA pathway, leading to Thr34 phosphorylation of DARPP-32 and subsequent CREB activation, revealing a distinct Wnt-5A/DARPP-32 signalling axis that inhibits breast cancer cell migration [14].

Following these early links to DDR1 modulation of signalling, additional studies have identified other mechanisms by which DDR1 can influence breast cancer cell behaviour. This includes transient regulation of CD9 cell-surface levels by type IV collagen [15], interaction with the non-receptor tyrosine kinase Syk to modulate epithelial migration [16], and downstream transcriptional repression of H-Ras/ZEB1 signalling, which reduces DDR1 expression and contributes to the epithelial-to-mesenchymal transition in triple-negative breast cancer cells [17]. DDR1 function is also modulated by membrane-type collagenases (MT1-, MT2-, and MT3-MMPs), which cleave DDR1 within its extracellular juxtamembrane region to attenuate collagen-induced activation [18]. In addition, DDR1 is implicated in chemoresistance and growth-promoting pathways, including induction of cyclooxygenase-2 (COX-2) expression [19], functional cross-talk with the insulin-like growth factor-I receptor (IGF-IR) that promotes proliferation and migration through PI3K/AKT-dependent DDR1 upregulation [20], and non-canonical DDR1 signalling through a TM4SF1/syntenin 2/PKCα/JAK2/STAT3 pathway that has been shown to sustain cancer stem cell traits and drive metastatic reactivation in lung, bone, and brain tumours [21].

Limited studies have investigated the prognostic role of DDR1 expression in breast cancer to explore links with clinical outcome. DDR1 expression has been shown to differ between lobular and ductal invasive carcinomas [22]. Several studies have determined DDR1 expression in relatively small cohorts of patients (*n* = 94–198) and have shown no link between DDR1 expression and the clinical outcome of patients [23,24,25]. In addition to breast cancer, there is evidence suggesting a role for DDR1 in ovarian cancer. In a cohort of 67 ovarian cancer patients, high DDR1 expression is linked with adverse disease-free survival [26]. To resolve uncertainty surrounding the prognostic significance of DDR1, this study sought to investigate DDR1 expression in both breast and ovarian cancer using large well-annotated clinical cohorts and define the transcriptomic consequences of DDR1 loss in cell line models of both cancer types.

## 2. Materials and Methods

### 2.1. Immunohistochemistry

Immunohistochemistry was carried out on both breast and ovarian tissue microarrays, with each constructed of single 0.6 mm cores from patient tumours. Cores were taken from representative invasive tumour regions identified by experienced breast and ovarian cancer histopathologists using haematoxylin and eosin-stained sections, which have been used in numerous previous studies [27,28,29,30,31]. Tissue microarray sections (4 µm thick) were deparaffinised and rehydrated through xylene, graded ethanol, and distilled water. Antigen retrieval was performed using sodium citrate buffer (pH 6.0). Slides were heated in a microwave using high power for 5 min, medium power for 5 min, followed by low power for 15 min. Staining was performed using a Novolink Polymer Detection kit (Leica, UK) according to the manufacturer’s instructions. Primary antibodies were incubated in tissue for one hour at room temperature (DDR1: Cell Signaling Technology, UK, 5583 1:50). Following staining, tissue was dehydrated in ethanol and fixed in xylene prior to mounting using DPX. All stained slides were scanned using a NanoZoomer digital pathology scanner (Hamamatsu Photonics, UK).

Cytoplasmic expression levels of DDR1 were determined using a semi-quantitative H-score approach, in which the proportion of positively stained tumour area was multiplied by the assigned intensity score ranging from 0 to 3 (absent, weak, moderate, or strong staining). Scoring was conducted by two independent assessors, blinded to each other’s scores and the clinical variables of the cohorts. Intraclass correlation coefficients were used to assess scoring reliability, with values greater than 0.7 considered to indicate good agreement between observers. This study is reported according to REMARK criteria [32].

### 2.2. Breast Cancer Patient Cohort

Tissue used to construct the breast cancer tissue microarray was obtained from patients treated at Nottingham University Hospitals between 1998 and 2006. Patients had either breast-conserving surgery or mastectomy, depending on clinical need and/or patient choice, and radiotherapy was given where clinically indicated. Clinical parameters including Nottingham Prognostic Index (NPI), oestrogen receptor (ER) status, and menopausal status were recorded before starting adjuvant therapy. ER-negative and premenopausal patients with an NPI score of 3.4 or greater received combination chemotherapy (cyclophosphamide, methotrexate, and 5-fluorouracil), whereas patients with an NPI score less than 3.4 did not receive adjuvant chemotherapy. ER-positive patients were treated with hormonal therapy. Disease-specific survival was measured from primary surgery to breast cancer-related death.

### 2.3. Ovarian Cancer Patient Cohort

Tissue used to construct the ovarian cancer tissue microarray was obtained from patients treated at Nottingham University Hospitals between 1991 and 2011. Patients were treated with adjuvant chemotherapy, with 63.6% receiving platinum-based chemotherapy, and resistance to chemotherapy was classified according to the Gynaecologic Oncology Group (GOG) into groups of refractory, resistant or sensitive. Clinicopathological data of patients was collected and included patient age, stage of the cancer, histological subtype, and grade, with grading achieved using the Shimizu–Silverberg grading system, in which tumours are given a score from 1 to 3 according to the degree of 3 parameters (architectural pattern, nuclear pleomorphism, and mitotic count). Overall survival was defined as the length between the start of treatment and date of death or last follow-up date. Progression-free survival was defined as the length between the start of treatment and clinical identification of recurrence or last follow-up date.

### 2.4. Statistics

Data analysis was performed using IBM SPSS Statistics version 29. Expression cut points were derived using X-tile software (version 3.6.1) with survival as the endpoint, applied consistently across cohorts [33]. Categorised protein expression levels and clinicopathological variables were evaluated using the Pearson chi-square test of association. The association between expression and clinical outcome was determined using Kaplan–Meier survival analysis with statistical differences determined using the log-rank test. A *p* value of less than 0.05 was considered statistically significant. Survival analyses within clinically defined subgroups (ER status) were exploratory and motivated by the established subtype-specific biology of DDR1; formal correction for multiple comparisons was not applied, and these analyses are reported as hypothesis-generating.

### 2.5. Cell Culture

Cell culture experiments were performed using the T47D breast cancer and the IGROV1 ovarian cancer cell lines, both purchased from American Type Culture Collection. Cells were cultured in high-glucose Dulbecco’s Modified Eagle’s Medium (Sigma, MA, USA), with 10% heat-inactivated iron-supplemented donor bovine serum (Gibco, NY, USA), and with 1% penicillin/streptomycin (Sigma). Cell line identity was verified every 24 months using short tandem repeat (STR) verification and cells were routinely monitored for mycoplasma infection.

### 2.6. DDR1 siRNA Knockdown

T47D and IGROV1 cells were subject to DDR1 knockdown using siRNA. Transfection was achieved using Lipofectamine 3000 in Opti-MEM reduced-serum medium (Thermo-Fisher, MA, USA) and 11.2nM siRNA (DDR1: Origene, MD, USA, SR319533A) or negative control siRNA (OriGene SR30004). Cell transfection occurred over 24 h, and knockdown was assessed at 24, 48, and 72 h post-transfection.

Gene knockdown was confirmed using Western blotting at multiple time-points using an Invitrogen Bolt Mini Gel system fitted with 4–12% Bis–Tris Plus gels. Protein lysates were prepared in Bolt LDS sample buffer supplemented with Bolt reducing agent and heat-treated at 100 °C for 5 min to ensure denaturation. Following electrophoresis, proteins were transferred to nitrocellulose membrane (Whatman, GE Healthcare, IL, USA) using Bolt transfer buffer containing 10% methanol. Membranes were subsequently blocked for 1 h in 3% (*w*/*v*) non-fat milk to reduce non-specific binding. Primary antibody incubation was performed overnight at 4 °C using antibodies against DDR1, DARPP-32 or β-actin (DDR1: Cell Signalling Technology (NE, USA), 5583 1:1000, β-actin: Abcam (UK), ab8226; 1:1000). Following antibody incubation, membranes were washed and incubated with the appropriate secondary antibodies: donkey anti-rabbit IgG (LI-COR, 926-32213; 1:10,000) or donkey anti-mouse IgG (LI-COR, 926-68072; 1:10,000) for 1 h at room temperature. Membranes were visualised using the Odyssey FC Imaging System (LI-COR) using Image Studio software (V4.1). 

### 2.7. RNA Sequencing

Total RNA extraction, mRNA library construction, and RNA sequencing were performed by Novogene UK. Libraries were prepared using poly-A enrichment and sequenced on Illumina NovaSeq (UK) platforms which utilised a paired-end 150 base pair sequencing strategy with data output greater than 20 million read pairs per sample. For data assessment, sequencing quality was assessed using FastQC (version 0.12.1), with adapter trimming and quality filtering performed using Trim Galore (version 0.6.7), and transcript abundance was quantified using Kallisto (version 0.50.0), which applies a pseudo-alignment approach [34]. Transcript identification and quantification were performed using the human reference genome GENCODE GRCh38 version 36 for transcript identification. Differential transcript expression analysis was assessed using DESeq2 (version 1.42.1), with Kallisto outputs imported via the Tximport package (1.30.1). All analyses were performed in RStudio 2025.05.1 build 513 [35]. Transcripts were filtered following multiple-testing correction, with significance defined as an adjusted *p* value < 0.05 and an absolute fold change greater than two. Functional and pathway enrichment analyses were carried out using Qiagen Ingenuity Pathway Analysis (IPA) (version 24.0.2).

### 2.8. Bioinformatic Assessments

To examine DDR1 knockdown-specific signalling between breast and ovarian cancer, we utilised a hub-centred protein–protein interaction (PPI) framework using the STRING database (version 11.5) and using a high confidence threshold (score ≥900). Significant differentially expressed transcripts from RNA-Seq were mapped to HGNC gene symbols using the Ensembl BioMart API. Unique and shared gene sets between cell lines were identified, with shared genes designated as “core” hubs. All candidate genes were mapped to STRING identifiers, and PPI networks were filtered to retain the top 600 nodes by connectivity. Hub-centred subgraphs were generated by selecting the highest-degree core hubs (top 8) and limiting the number of neighbours per hub (max 10) to highlight key regulatory nodes while reducing network complexity with data plotted using an organic Fruchterman–Reingold layout via ggraph/igraph.

For unsupervised analysis of DDR1 signalling, ER-positive breast cancer patients were selected with complete immunohistochemistry data available for DARPP-32 (cytoplasmic and nuclear), DARPP-32 Threonine-34 phosphorylation (cytoplasmic and nuclear), PKA, PP1 (cytoplasmic and nuclear), CDK5 (cytoplasmic and nuclear), and CREB1 in the same breast cancer patient cohort (*n* = 304) [36,37,38]. Protein H-scores were z-scored and subjected to principal component analysis (PCA) using the prcomp function in R; the first principal component (PC1), representing coordinated pathway activity, was used for downstream survival analyses in SPSS as described previously. All analysis scripts are available on GitHub (https://github.com/sarahstorr/DDR1, accessed on 13 May 2026).

## 3. Results

### 3.1. DDR1 Expression in Breast Cancer

**Figure 1:**
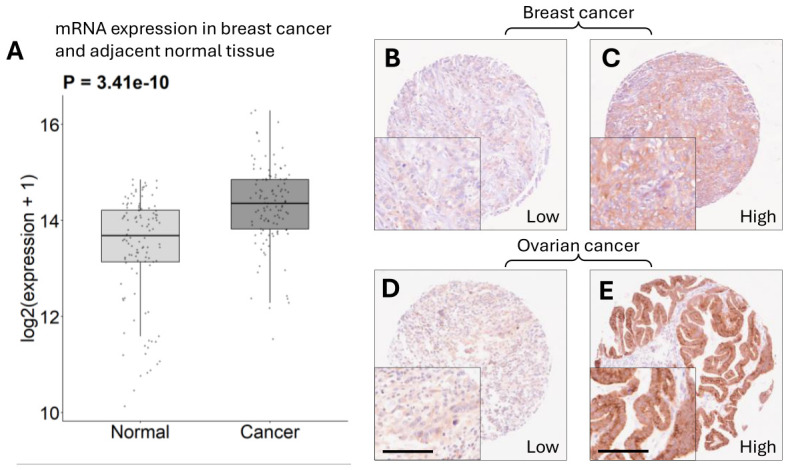
(**A**) DDR1 mRNA expression in cancer and matched adjacent normal tissue in breast cancer. (**B**) Low DDR1 expression in breast cancer, (**C**) high expression of DDR1 in breast cancer, (**D**) low DDR1 expression in ovarian cancer, (**E**) high expression of DDR1 in ovarian cancer. Photomicrographs are shown at 10× magnification with a 20× magnification inset box, where the scale bar represents 100 µm.

A comparison of *DDR1* mRNA expression levels between matched tumour and adjacent normal tissues in the TCGA breast cancer cohort revealed significantly increased DDR1 expression in tumour tissue (*p* < 0.001) (Figure 1A). DDR1 protein expression was determined using immunohistochemistry in tumour tissues from 1416 patients with breast cancer. Staining was predominantly cytoplasmic, with representative staining shown in Figure 1B,C. The median H-score for cytoplasmic DDR1 expression was 100, ranging from 0 to 270. X-tile generated a cut point of 120, with 38.3% (543/1416) demonstrating high staining.

High DDR1 expression was significantly associated with clinicopathological criteria, including higher-grade tumours (χ^2^ = 35.33, d.f. = 2, *p* < 0.001), marked nuclear pleomorphism (χ^2^ = 39.74, d.f. = 2, *p* < 0.001), high mitotic index (χ^2^ = 46.38, d.f. = 2, *p* < 0.001), tumours larger than 2cm (χ^2^ = 9.77, d.f. = 1, *p* = 0.002), presence of lymphovascular invasion (χ^2^ = 6.88, d.f. = 1, *p* = 0.009), intrinsic molecular subtype (χ^2^ = 48.72, d.f. = 3, *p* < 0.001), ER-positive tumours (χ^2^ = 4.36, d.f. = 1, *p* = 0.037), and HER2-positive tumours (χ^2^ = 74.46, d.f. = 1, *p* < 0.001) (Table 1).

**Figure 2:**
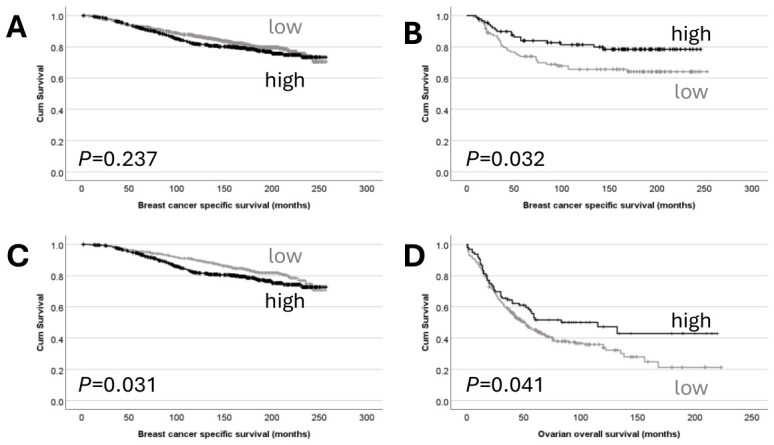
Kaplan–Meier analysis of the effect of DDR1 expression in patient cohorts where low (grey line) and high (black line) expression is shown. (**A**) High (*n* = 543) and low (*n* = 873) DDR1 protein expression in the total breast cancer cohort. (**B**) High (*n* = 90) and low (*n* = 110) DDR1 protein expression in ER-negative breast cancers. (**C**) High (*n* = 453) and low (*n* = 763) DDR1 expression in ER-positive breast cancers. (**D**) High (*n* = 96) and low (*n* = 343) DDR1 expression in the total ovarian cancer cohort.

Cytoplasmic DDR1 expression was not significantly associated with disease-specific survival in the whole patient cohort (Figure 2A). In ER-negative breast cancer, low cytoplasmic DDR1 expression was significantly associated with worse survival (*p* = 0.032) (Figure 2B). In contrast, high cytoplasmic DDR1 expression was significantly associated with adverse disease-specific survival in ER-positive breast cancer (*p* = 0.031) (Figure 2C). In multivariate Cox-regression models of survival, DDR1 remained associated with disease-specific survival of ER-negative patients (hazard ratio (HR) = 0.514, 95% confidence interval (CI) = 0.290–0.911, *p* = 0.023), when tumour size, grade, lymphovascular invasion, and lymph node stage were included in the model. In ER-positive patients, DDR1 expression was not associated with disease-specific survival when the same variables were included in the model (HR = 1.077, 95% CI = 0.815–1.423, *p* = 0.603).

### 3.2. DDR1 Expression in Ovarian Cancer

A comparison of *DDR1* mRNA expression between matched adjacent normal and tumour ovarian tissues could not be performed as matched normal samples are not available within the TCGA ovarian cancer cohort. DDR1 protein expression was determined using immunohistochemistry in tumour tissues from 450 patients with ovarian cancer. Staining was predominantly cytoplasmic with representative staining shown in Figure 1D,E. The median H-score for cytoplasmic DDR1 expression was 180, ranging from 0 to 280. X-tile generated a cut point of 200, with 21.6% (97/450) demonstrating high staining.

High DDR1 expression was significantly associated with tumour histology (χ^2^ = 20.15, d.f. = 6, *p* = 0.003). No other associations were observed between DDR1 expression and clinicopathological criteria (Table 2). High DDR1 expression was associated with improved overall survival of ovarian cancer patients (*p* = 0.041) (Figure 2D). In multivariate Cox-regression models of survival, DDR1 expression remained associated with survival (HR = 0.694, 95% CI = 0.501–0.962, *p* = 0.028) when tumour grade, FIGO stage, and histology were included in the model.

### 3.3. DDR1 Knockdown in Breast and Ovarian Cancer Cell Lines

**Figure 3:**
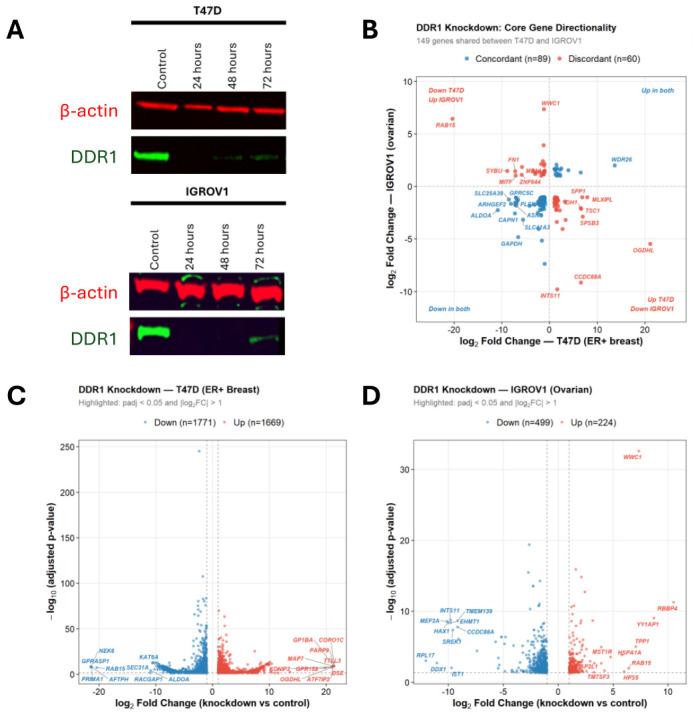
(**A**) Western blot time-course analysis of *DDR1* knockdown in T47D and IGROV1 cell lines. DDR1 is shown in green, and β-actin is shown in red as a loading control. (**B**) Scatter plot illustrating directionality of gene expression changes across the 149 shared genes differentially expressed in *DDR1* knockdown in T47D and IGROV1. Volcano plots of differentially expressed genes in *DDR1* knockdown T47D (**C**) and IGROV1 (**D**) cell lines. Genes with a Benjamini–Hochberg adjusted *p* value < 0.05 and |log_2_ fold change| > 1 are highlighted in red (upregulated) or blue (downregulated). The top differentially expressed genes ranked by fold change magnitude are labelled.

To investigate transcriptomic changes resulting from altered DDR1 expression, *DDR1* was subject to knockdown in T47D breast and IGROV1 ovarian cancer cells using siRNA. Western blot analysis demonstrated a reduction in DDR1 protein levels of more than 70% in T47D cells, 24 h after transfection, and 90% in IGROV1 cells at the same time-point (Figure 3A). Knockdown cells were then subjected to RNA sequencing.

**Figure 4:**
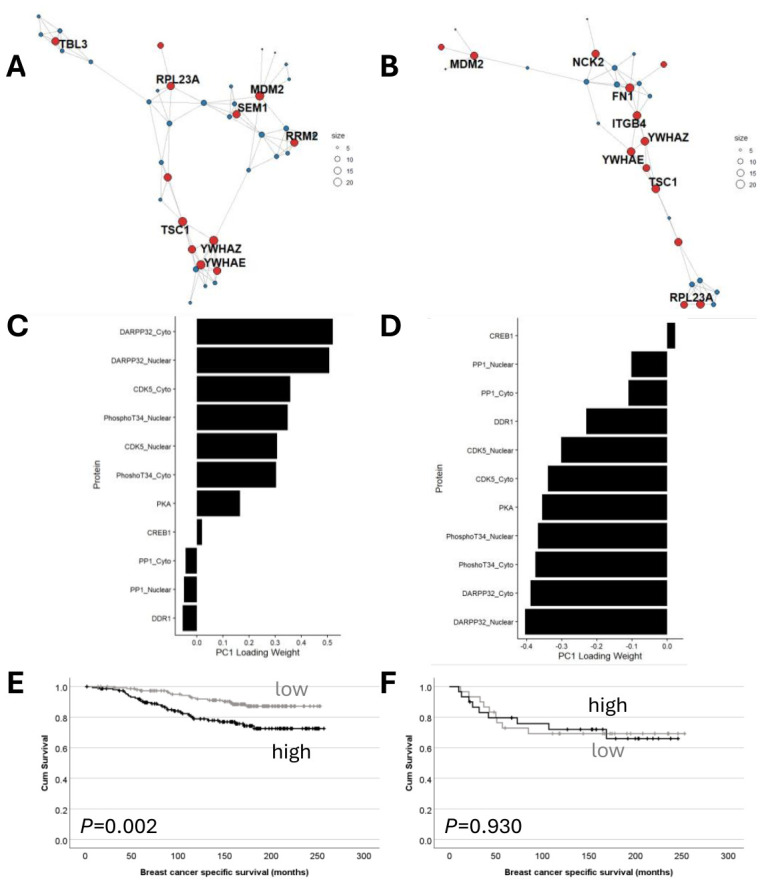
(**A**) Hub-centred STRING networks of *DDR1* knockdown T47D cells and IGROV1 cells (**B**). Red indicates shared core hubs and blue indicates cell line-specific genes. Node size corresponds to the number of interactions, highlighting highly connected proteins. Only the top core hubs are labelled for clarity, while less connected core nodes remain unlabelled. Edges represent high-confidence protein–protein interactions (STRING score ≥900). Loading weight of each protein to PC1, where positive loading weight corresponds to proteins that increase when PC1 increases, whereas negative loading weight indicates an inverse association with the axis; shown in ER-positive cohort (**C**) and ER-negative cohort (**D**). Kaplan–Meier analysis of the effect of PC1 (median stratified) in patient cohorts, where low (grey line) and high (black line) is shown in the ER-positive cohort (**E**) and the ER-negative cohort (**F**).

In T47D cells, *DDR1* knockdown resulted in the identification of 3647 differentially expressed transcripts, mapping to 3440 genes, when compared with control cells (Figure 3C and Supplementary File S1). In contrast, 770 differentially expressed transcripts, mapping to 723 genes, were identified in IGROV1 cells (Figure 3D and Supplementary File S2). Overall, 149 overlapping genes formed the shared core network, providing a functional backbone linking *DDR1*-dependent transcriptional changes across the two cell lines (Figure 3B and Supplementary File S3); this corresponded to 44 overlapping unique transcripts. Gene Ontology analysis of the overlapping core genes revealed enrichment for only three molecular functions: sulphur compound binding, heparin binding, and scaffold protein binding. The hub-centred network analysis identified several core genes shared between T47D and IGROV1 cells (Figure 4A and 4B, respectively). Major core hubs identified across both cell lines included *MDM2*, *YWHAZ*, *YWHAE*, and *TSC1*, underscoring conserved regulatory nodes in DDR1 signalling. Cell line-specific genes were compared to the shared core to contextualise their interactions within a stable network framework. This approach allowed identification of connections that are either conserved across both cell lines or uniquely associated with *DDR1* knockdown in a particular context.

### 3.4. Pathway-Level Signalling Axis Investigation in ER-Positive and -Negative Breast Cancer Subgroups

As some DDR1 linked proteins were available in the breast cancer cohort, additional pathway analysis was performed (DARPP-32, PKA, PP1, and CREB1) [36,37,38]. In the ER-positive cohort with complete data available (*n* = 304), PCA identified a dominant signalling axis (PC1) driven primarily by cytoplasmic and nuclear DARPP-32 (Figure 4C). Lower PC1 scores were associated with worse disease-specific survival in ER-positive patients (*p* = 0.01) (Figure 4E), indicating that coordinated pathway activity, rather than DDR1 expression alone, defines prognostic variation within this subgroup. In contrast, in the ER-negative subgroup with complete data available (*n* = 60), PCA revealed a signalling axis with mostly negative loading weights (Figure 4D). Kaplan–Meier survival analysis indicated no association with disease-specific survival (*p* = 0.93) (Figure 4F).

## 4. Discussion

DDR1 is a collagen-activated receptor tyrosine kinase that plays an important role in epithelial cell regulation with context-dependent roles in cancer that vary by tumour type. In this study, we investigated large, well annotated cohorts of breast and ovarian cancer patients to define the relationship between DDR1 expression and clinical outcome.

In breast cancer, DDR1 expression was not associated with overall survival in the total patient cohort. However, stratification by ER status revealed opposing associations; low cytoplasmic DDR1 expression was significantly associated with worse patient outcome in ER-negative breast cancer, and high DDR1 expression was significantly associated with worse patient outcome in ER-positive breast cancer. Consistent with the association observed in ER-negative breast cancer, high DDR1 expression was associated with improved overall survival in ovarian cancer patients. Together, these findings indicate that DDR1 expression is associated with patient outcome in a context- and subtype-specific manner.

Previous studies investigating DDR1 expression in breast cancer have been conducted in relatively small cohorts of patients and have not demonstrated a link between DDR1 protein expression and clinical outcome [23,24,25]. Two of these studies assessed the relationship between DDR1 expression and clinicopathological variables and found no significant associations [24,25]. Interestingly, one publication demonstrated that low DDR1 expression combined with high DDR2 expression was associated with triple-negative breast cancers, and this specific expression subgroup (DDR1 low/DDR2 high) was also associated with clinical outcome [25]. Notably, despite their limited cohort sizes, these studies report patterns of association that are broadly consistent with those observed in the current study. In ovarian cancer (*n* = 67), high DDR1 expression has been associated with adverse disease-free survival [26], which contrasts with the findings presented within this study. A study has, however, demonstrated that *DDR1* mRNA expression is lower in epithelial ovarian cancer cell lines categorised as mesenchymal-like compared to those categorised as epithelial-like, suggesting a loss of DDR1 as cells undergo the epithelial-to-mesenchymal transition; in addition, lower DDR1 was also reported in patient samples with a mesenchymal molecular subtype [39].

To generate hypotheses regarding the basis for the divergent associations observed, transcriptomic analyses were performed following *DDR1* knockdown in breast and ovarian cancer cell lines. These analyses were intended to identify candidate genes and pathways associated with reduced DDR1 expression. DDR1 expression was successfully reduced by over 70% in both T47D and IGROV1 cell lines, with 3647 and 770 differentially expressed transcripts identified, respectively. Of these, 149 genes were shared between the two cell lines, indicating substantial transcriptional divergence following *DDR1* knockdown. Genes shared by IGROV1 and T47D included key regulators of signalling, metabolism, and cytoskeletal organisation, such as *YWHAE*, *NCK2*, *FN1*, and *ITGB4*. DDR1 has previously been shown to interact directly with both YWHAE and NCK2 under flow conditions or in a collagen-dependent manner, respectively [40,41]. Fibronectin (*FN1*) and *ITGB4* are established mediators of extracellular signalling and cellular migration and their identification may indicate a role for DDR1 in regulating extracellular matrix adhesion in breast and ovarian cancer. Further Gene Ontology analysis demonstrated significant enrichment of epithelial cell migration pathways in both cell lines, suggesting a conserved role for DDR1 in regulating epithelial motility. A limitation of this study is the reliance on a single cell line for each cancer type, which only captures part of the biological heterogeneity of breast and ovarian cancer. Future work will extend these analyses to additional cell models, including subtype-representative lines, to validate and broaden the transcriptional changes identified. Furthermore, targeted validation of differentially expressed genes (for example, by qPCR or protein-based approaches) and assays to explore phenotypic changes resulting from DDR1 knockdown are required to confirm and functionally contextualise the transcriptional changes identified.

A range of data on DDR1-associated signalling components were available within the breast cohort and this enabled a broader pathway-level analysis and identified a dominant signalling axis that was principally driven by cytoplasmic and nuclear DARPP-32 expression in ER-positive tumours. Reduced levels of this signalling axis were significantly associated with adverse disease-specific survival, suggesting that reduced activity of the signalling programme rather than DDR1 alone is more important in ER-positive disease prognosis. This is in contrast to ER-negative tumours, where the signalling axis was not associated with disease-specific survival, indicating that DDR1 signalling coordination may be less relevant in this subgroup. Interestingly, DDR1 has been shown to suppress breast cancer cell migration in vitro through the formation of a functional complex with DARPP-32 [13]. In addition, we have previously demonstrated that low DARPP-32 protein expression is associated with adverse survival of ER-positive patients [36].

Although the present study is descriptive, the differentially expressed genes and interactome data allow the formation of a tentative working model. Across both breast and ovarian cells, DDR1 knockdown altered a shared core that included recognised DDR1 interactors (*YWHAE* and *NCK2*) [40,41] alongside mediators of extracellular matrix adhesion and motility (*FN1* and *ITGB4*), with Gene Ontology analysis implicating epithelial cell migration in both lines. We therefore hypothesise that a conserved function of DDR1 in these tumours is the coordination of adhesion- and migration-related signalling through scaffold and adaptor proteins, consistent with its established role as a collagen-activated regulator of epithelial behaviour. In addition, we observe context-specific programmes; in ER-positive breast cancer, the previously reported DDR1 interaction with DARPP-32 links DDR1 to a coordinated DARPP-32 centred signalling axis encompassing PKA, PP1, CDK5, and CREB1 [13]. In our cohort, lower coordinated activity of this axis (PC1) was associated with adverse disease-specific survival in ER-positive patients.

When considering patient cohort analyses and in vitro transcriptomic profiling, this study links DDR1 expression patterns with clinical outcome in breast and ovarian cancer and provides descriptive, hypotheses-generating transcriptomic data that highlight candidate molecular processes that are potentially relevant to these associations. Survival associations should be interpreted cautiously as subgroup analyses were not corrected for multiple comparisons, and several associations were of borderline significance. Independent validation in additional patient cohorts is required. Differential expression was determined from three biological replicates per condition and assessed using DESeq2 with correction for multiple testing. Two of the shared differentially expressed genes, YWHAE and NCK2, have previously been documented to interact with DDR1. The complete raw and processed sequencing data have been deposited in public repositories (GEO: GSE312892; SRA: PRJNA1377570) and are available for independent inspection. Further functional and mechanistic studies are needed to understand the context- and subtype-specific differences observed. Collectively, these findings support a role for DDR1 in modulating tumour behaviour in a context-dependent manner.

In summary, the expression of DDR1 shows tumour- and subtype-specific associations with clinical outcome in breast and ovarian cancer, which align with distinct transcriptional programmes. These findings point to a context-dependent role for DDR1 in tumour biology and support further evaluation as a biomarker across cancer types.

## Figures and Tables

**Table 1:** Associations between DDR1 expression determined using immunohistochemistry with clinicopathological variables in breast cancer. The *p* values are resultant from the Pearson χ^2^ test of association. ER is oestrogen receptor and PgR is progesterone receptor.

		DDR1 Expression		
		Low	High	*p* Value
Lymph node stage				
	1	545 (38.5%)	328 (23.2%)	0.142
	2	255 (18.0%)	153 (10.8%)	
	3	72 (5.1%)	62 (4.4%)	
Tumour grade				
	1	156 (11.0%)	66 (4.7%)	<0.001
	2	407 (28.7%)	198 (14%)	
	3	310 (21.9%)	279 (19.7%)	
Tumour size				
	Less than 2 cm	563 (39.8%)	305 (21.5%)	0.002
	2 cm or greater	310 (21.9%)	238 (16.8%)	
Tubules				
	1	69 (4.9%)	32 (2.3%)	0.358
	2	262 (18.5%)	168 (11.9%)	
	3	542 (38.3%)	343 (24.2%)	
Pleomorphism				
	1	15 (1.1%)	10 (0.7%)	<0.001
	2	320 (22.6%)	113 (8%)	
	3	538 (38.0%)	420 (29.7%)	
Mitotic index				
	1	498 (35.2%)	219 (15.5%)	<0.001
	2	174 (12.3%)	115 (8.1%)	
	3	201 (14.2%)	209 (14.8%)	
Lymphovascular invasion				
	Absent	645 (45.6%)	366 (25.8%)	0.009
	Present	228 (16.1%)	177 (12.5%)	
Molecular subclass				
	Luminal A	393 (32.3%)	163 (13.4%)	<0.001
	Luminal B	252 (20.7%)	225 (18.5%)	
	HER2 Enriched	9 (0.7%)	22 (1.8%)	
	TNBC	93 (7.6%)	60 (4.9%)	
ER status				
	Negative	110 (7.8%)	90 (6.4%)	0.037
	Positive	763 (53.9%)	453 (32.0%)	
PgR status				
	Negative	283 (20.1%)	193 (13.7%)	0.208
	Positive	585 (41.6%)	345 (24.5%)	
HER2 status				
	Negative	831 (58.7%)	439 (31.0%)	<0.001
	Positive	42 (3.0%)	104 (7.3%)	
Patient age				
	Less than 50 years	259 (18.3%)	169 (11.9%)	0.562
	50 years or greater	614 (43.4%)	374 (26.4%)	

**Table 2:** Associations between DDR1 protein expression determined using immunohistochemistry with clinicopathological variables in ovarian cancer. The *p* values are resultant from the Pearson χ^2^ test of association.

		DDR1 Expression		
		Low	High	*p* Value
FIGO stage	1	120 (27.1%)	40 (9%)	0.167
	2	43 (9.7%)	8 (1.8%)	
	3	163 (36.8%)	38 (8.6%)	
	4	21 (4.7%)	10 (2.3%)	
Grade	1	31 (6.9%)	5 (1.1%)	0.520
	2	53 (11.8%)	15 (3.3%)	
	3	269 (59.9%)	76 (16.9%)	
Chemotherapy response	Sensitive	193 (63.1%)	60 (19.3%)	0.093
	Resistant	46 (15%)	7 (2.3%)	
Histology	High-grade serous	213 (47.3%)	67 (14.9%)	0.003
	Mucinous	39 (8.7%)	3 (0.7%)	
	Endometrioid	35 (7.8%)	16 (3.6%)	
	Clear-cell carcinoma	41 (9.1%)	1 (0.2%)	
	Low-grade serous	14 (3.1%)	7 (1.6%)	
	Borderline serous	10 (2.2%)	3 (0.7%)	
	Borderline mucinous	1 (0.2%)	0 (0%)	
Age	60 years or younger	167 (37.1%)	47 (10.4%)	0.842
	61 years or greater	186 (41.3%)	50 (11.1%)	

## Data Availability

The processed gene expression data have been deposited in the Gene Expression Omnibus (GEO) under accession GSE312892. Raw sequencing data are available in the Sequence Read Archive (SRA) under the associated BioProject PRJNA1377570. All analysis scripts are available on GitHub (https://github.com/sarahstorr/DDR1, accessed on 13 May 2026).

## Supplementary Materials

The following supporting information can be downloaded at https://www.mdpi.com/article/10.3390/genes17070760/s1: Supplementary File S1: DDR1 knockdown of 3647 differentially expressed transcripts, mapping to 3440 genes, in T47D cells. Supplementary File S2: 770 differentially expressed transcripts, mapping to 723 genes identified in IGROV1 cells. Supplementary File S3: 149 overlapping genes across the two cell lines.
